# Granulomatous Lymphocyte Interstitial Lung Disease: A Rare Complication of Common Variable Immunodeficiency Managed With Azathioprine and Rituximab

**DOI:** 10.7759/cureus.59399

**Published:** 2024-04-30

**Authors:** Ali Khreisat, Vickie Xin, Christopher Dado

**Affiliations:** 1 Internal Medicine, Corewell Health William Beaumont University Hospital, Royal Oak, USA; 2 Pulmonary and Critical Care Medicine, Corewell Health William Beaumont University Hospital, Royal Oak, USA

**Keywords:** noncaseating granuloma, autoimmune lung disorder, granulomatous lymphocytic interstitial lung disease, rapidly progressive ild, common variable immunodeficiency (cvid)

## Abstract

Granulomatous lymphocytic interstitial lung disease (GL-ILD) is a rare, non-infectious pulmonary manifestation of common variable immunodeficiency (CVID). Diagnosing and managing GLILD remains challenging due to its poorly understood pathogenesis and high mortality. We present a complex case of a young female with CVID associated with lung and spinal cord involvement managed with azathioprine and rituximab.

## Introduction

Common variable immunodeficiency (CVID) is a primary immunodeficiency disorder characterized by failure of B-lymphocytic cell maturation, leading to hypogammaglobulinemia, recurrent sinopulmonary infections, autoimmune disorders, and malignancy [1]. However, pulmonary infectious and neoplastic complications are the most common challenges in patients with CVID. Granulomatous lymphocytic interstitial lung disease (GL-ILD) is one of the non-infectious complications associated with a poor prognosis and diagnostic and therapeutic difficulties [2].

GL-ILD is defined by the British Lung Foundation as a ‘’distinct clinico-radio-pathological" interstitial lung disease occurring in patients with common variable immunodeficiency disorders associated with a lymphocytic infiltrate and/or granuloma in the lung, and in whom other conditions have been considered and where possible excluded’’ [3]. The pathophysiology of GL-ILD is poorly understood; however, it is speculated that it is an outcome of an immune dysregulation phenomenon, as it was observed that patients with CVID and GL-ILD have a higher incidence of autoimmune complications [4].

The diagnosis of GL-ILD is often delayed due to nonspecific imaging findings on CT scans, such as ground-glass opacities, lung nodules, and mediastinal lymphadenopathy [5]. A surgical lung biopsy showing noncaseating granuloma, organizing pneumonia, peribronchiolar, and interstitial lymphocytic hyperplasia is often warranted to establish a diagnosis [6].

## Case presentation

**Figure 1:**
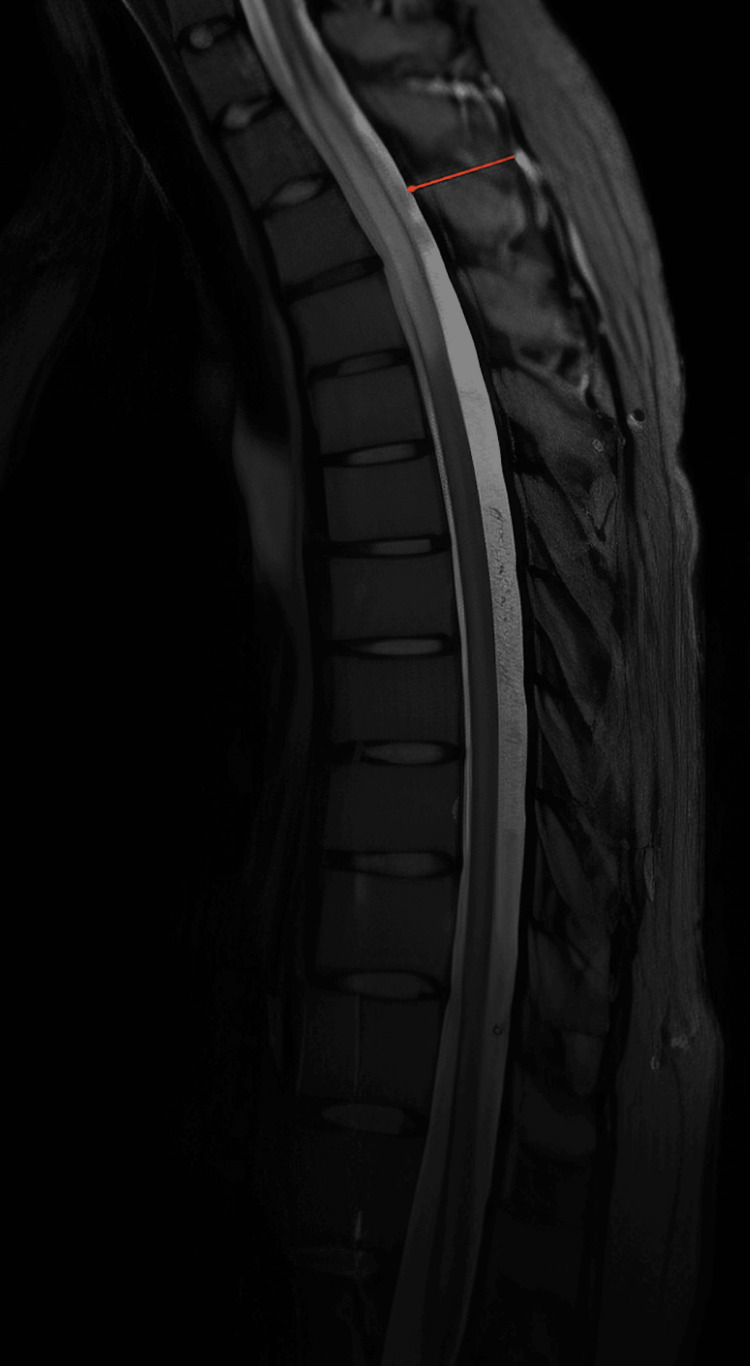
The patient's thoracic spine magnetic resonance imaging showing focal long segment area of abnormal hyperintensity in the thoracic spinal cord with cord swelling at the T1 through T5 levels with enhancement (red arrow).

**Figure 2:**
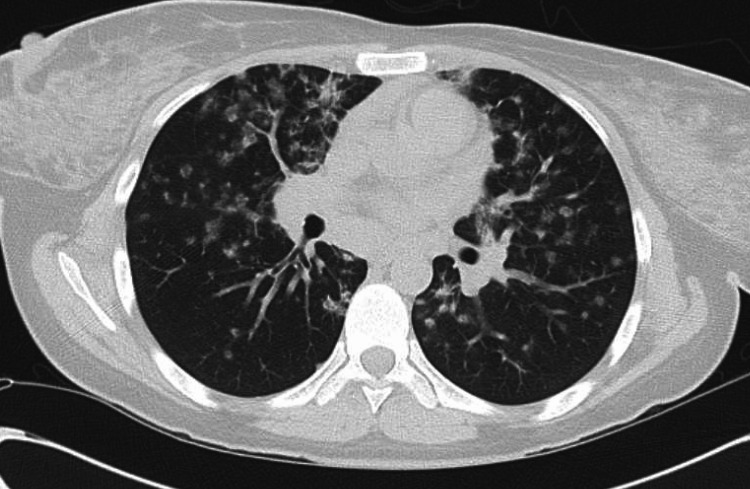
The patients initial chest computed tomography showing numerous pulmonary nodules with ground-glass appearance along with bilateral hilar lymphadenopathies.

A 39-year-old female with a medical history of immune thrombocytopenia (ITP), vitiligo, and CVID on weekly subcutaneous immunoglobulin injections initially presented to the hospital with a one-year history of progressive right leg numbness and gait imbalance. Physical examination showed decreased pinprick sensation and loss of proprioception in the bilateral lower extremities. Laboratory workups were largely unremarkable, including complete blood count (CBC), chemistry panel, immunoglobulins, vitamin B12 level, heavy metal testing, and antinuclear antibody levels. Spine MRI showed a hyperintense T2 signal within the upper thoracic spinal cord with enhancement (Figure 1). CSF analysis showed no oligoclonal bands, and the protein and cell count were normal. Although the patient was asymptomatic from a pulmonary standpoint, a chest X-ray showed bilateral hilar and mediastinal lymphadenopathy, along with numerous bilateral ground-glass opacities on chest CT (Figure 2). She was initially diagnosed with sarcoidosis as the unifying diagnosis for her pulmonary and neurologic imaging findings. Bronchoscopy with bronchoalveolar lavage was nondiagnostic. Mediastinal lymph node biopsies obtained through mediastinoscopy showed no granulomatous disease or malignancy. She was started on intravenous dexamethasone 4 mg twice daily for three days with improvement in her neurologic symptoms, and she was later discharged on a four-week taper course of prednisone starting at 40 mg daily.

**Figure 3:**
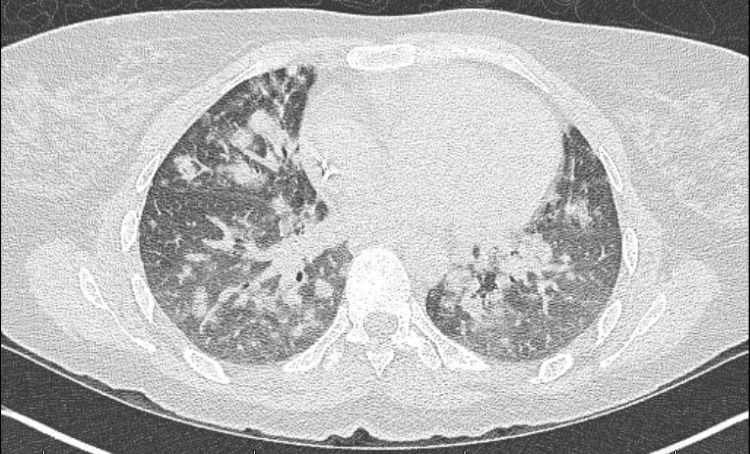
The patients high-resolution chest computed tomography scan showing mid and lower lobe predominant peribronchovascular ground-glass and consolidative nodular opacities.

**Figure 4:**
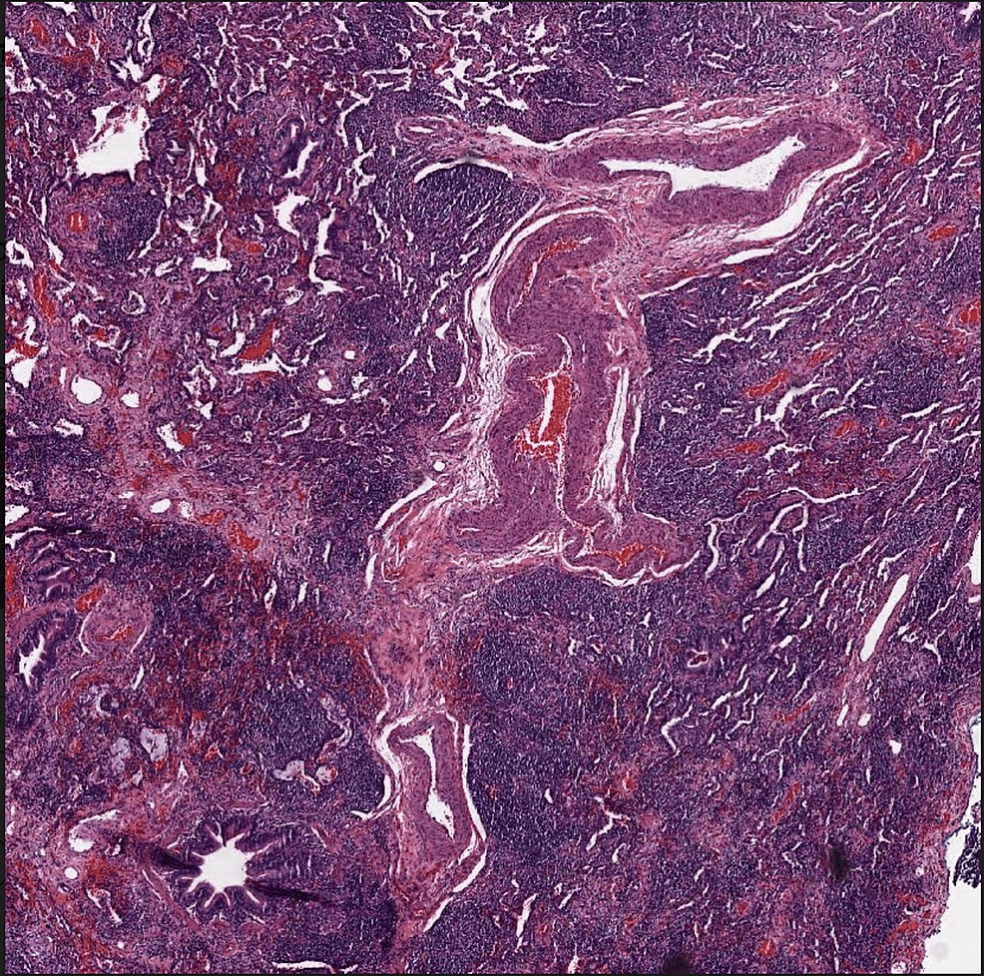
H&E-stained lung biopsy at 40× magnification showing interstitial expansion by variably dense inflammatory infiltrate, composed primarily of lymphocytes with scattered non necrotizing granulomas. The interstitial abnormality is cantered primarily around terminal airways. H&E: hematoxylin and eosin.

**Figure 5:**
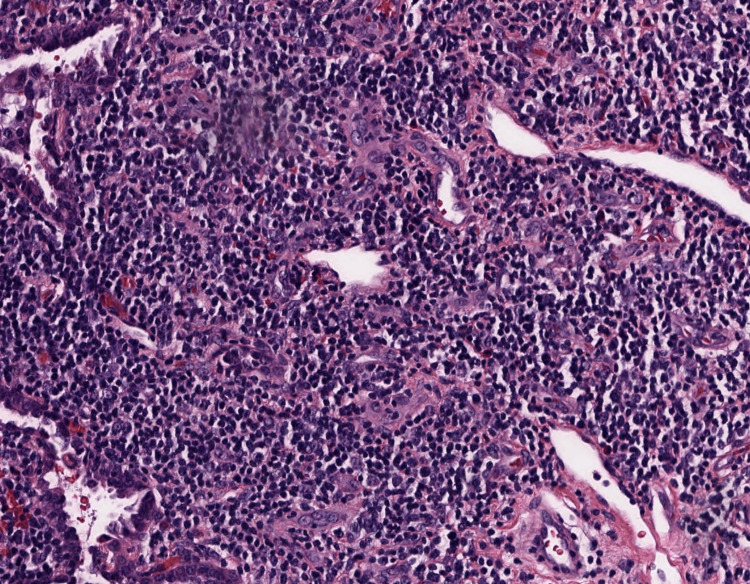
H&E stained lung biopsy slide at 200× magnification showing extensive lymphocytic interstitial infiltrates and scattered non-caseating granulomas around terminal airways consistent with GL-ILD.

The patient followed up closely with her outpatient rheumatologist; her neurological symptoms were modestly controlled with weekly 20 mg intramuscular methotrexate after her prednisone was tapered off. Annual pulmonary function testing and a high-resolution chest CT scan showed mild restrictive lung pathology and mid-lower lobe predominant peribronchovascular ground glass and consolidative nodular opacities, respectively (Figure 3). The patient was asymptomatic from a pulmonary standpoint and not hypoxic. The surgical pathology of a wedge biopsy obtained through video-assisted thoracoscopic surgery showed chronic interstitial pneumonia, characterized by interstitial expansion by a variably dense inflammatory infiltrate comprised mainly of lymphocytes with scattered non-necrotizing granulomas. The interstitial abnormality is centered primarily around terminal airways (Figures 4-5). These changes are complicated by patchy organizing pneumonia and focal areas of interstitial collagen deposition. Overall, this combination of features qualifies for the diagnosis of granulomatous-lymphocytic interstitial lung disease in the setting of the patient's known CVID.

**Figure 6:**
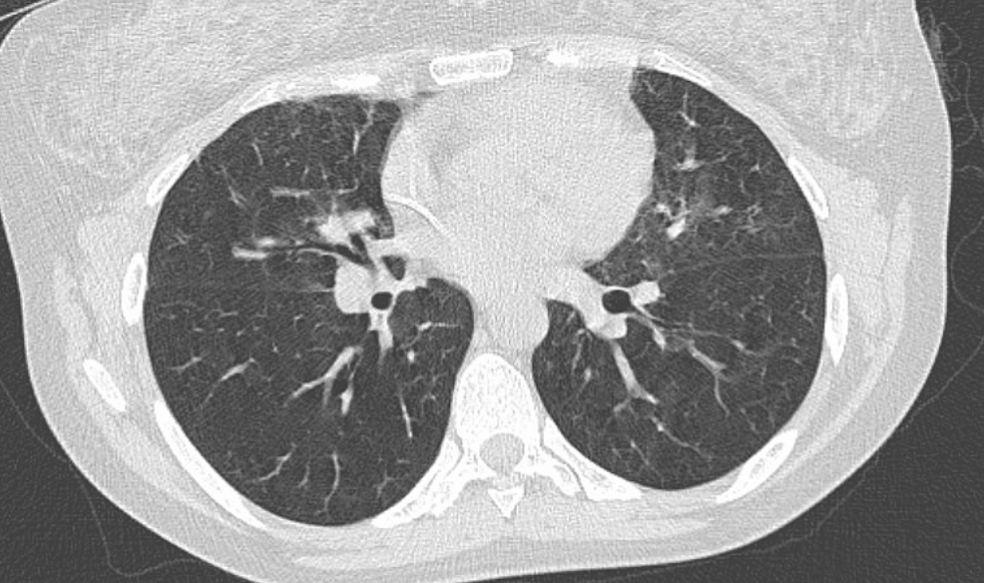
High-resolution chest computed tomography three months after starting rituximab and azathioprine showing significant interval decrease in mid-lower lung lobe patchy ground-glass opacities.

The etiology of her neurological symptoms was more in favor of the granulomatous myelitis seen in CVID. She was started on pulse therapy, followed by taper steroid therapy. The multidisciplinary team decided to proceed with rituximab 375 mg/m^2^ weekly for four weeks, then every six months for two years, and azathioprine 1 mg/kg daily, taking into account that this regimen has acceptable central nervous system penetration as a steroid-sparing therapy. The patient has maintained clinical recovery of her neurological symptoms and significant interval radiological improvement on follow-up high-resolution chest tomography and pulmonary function testing (Figure 6).

## Discussion

GL-ILD is a rare and complex complication of CVID, and its pathogenesis and diagnostic criteria are yet to be fully established. One of the most common non-infectious complications is chronic lung disease. While immunoglobulin therapy has reduced morbidity and mortality due to infection, the presence of non-infectious complications is associated with poor prognosis and quality of life [7].

Approximately 10-20% of those with CVID are diagnosed with GL-ILD, and it is associated with worse clinical outcomes [8]. Current guidelines recommend that patients obtain a chest CT and pulmonary function testing early in diagnosing CVID to establish a baseline. Once there is suspicion of GL-ILD, histology is the current gold standard for achieving a diagnosis. On histology, one should see noncaseating granulomas accompanied by peribronchiolar lymphoid infiltrations. These are distinct from the granulomas seen in sarcoid since they are not perilymphatic. In addition, follicular bronchiolitis and lymphoid interstitial pneumonia (LIP) seen in GL-ILD are not observed in sarcoidosis [9].

A multicenter retrospective cross-sectional study was completed, and its purpose was to investigate clinical, laboratory, and radiological parameters that may aid in diagnosing CVID patients who have GL-ILD. It concluded that a combination of splenomegaly, autoimmune cytopenia, DLCO, and increased CD21lo B cells is a promising criterion for early recognition of GL-ILD in CVID patients. The study emphasizes the difficulty in establishing a diagnosis, particularly when tissue diagnosis and the risks associated with obtaining a lung biopsy may outweigh the benefits in some cases [7].

Treatment of GL-ILD also poses challenges. There are very few controlled studies in which agents are successful in the treatment of GL-ILD. A survey conducted via a United Kingdom consortium of immunologists, chest physicians, radiologists, and pathologists interested in GL-ILD exposes the gaps in understanding the nuances of treatment. For example, the study demonstrated there was consensus that optimization of immunoglobulin therapy should be a standard protocol before initiating further specific treatment of GL-ILD; however, there was no consensus on whether a patient would benefit from maintaining a higher immunoglobulin trough level [3]. Ninety percent of those interviewed agreed that first-line treatment should be with corticosteroids, and at least 80% had a consensus that the second-line agents were azathioprine, rituximab, and mycophenolate [10]. Another issue regarding treatment is how the GL-ILD treatment response should be assessed and maintained. The survey demonstrated consensus that treatment response could be evaluated with an improvement in symptoms, lung function testing, and a change in CT appearance [3]. More studies are needed to assess the need for and choice of maintenance therapy in a clinically stable disease compared to clinical observation of treatment.

## Conclusions

GL-ILD is a unique entity and should be a physician's differential diagnosis for CVID patients with pulmonary complaints. This case report highlights the challenges of diagnosing and treating GL-ILD. Not only had this patient originally received a diagnosis of neurosarcoidosis, but the choice of treatment for her was also unique, given the concurrent pulmonary and neurological findings. Further randomized controlled trials are needed to investigate the outcome of steroid-sparing therapy agents in patients with GL-ILD.
